# Data-driven clustering of combined Functional Motor Disorders based on the Italian registry

**DOI:** 10.3389/fneur.2022.987593

**Published:** 2022-11-28

**Authors:** Giovanni Mostile, Christian Geroin, Roberto Erro, Antonina Luca, Enrico Marcuzzo, Paolo Barone, Roberto Ceravolo, Sonia Mazzucchi, Andrea Pilotto, Alessandro Padovani, Luigi Michele Romito, Roberto Eleopra, Carlo Dallocchio, Carla Arbasino, Francesco Bono, Pietro Antonio Bruno, Benedetta Demartini, Orsola Gambini, Nicola Modugno, Enrica Olivola, Laura Bonanni, Alberto Albanese, Gina Ferrazzano, Rosa De Micco, Maurizio Zibetti, Giovanna Calandra-Buonaura, Martina Petracca, Francesca Morgante, Marcello Esposito, Antonio Pisani, Paolo Manganotti, Fabrizio Stocchi, Mario Coletti Moja, Ilaria Antonella Di Vico, Lucia Tesolin, Francesco De Bertoldi, Tommaso Ercoli, Giovanni Defazio, Mario Zappia, Alessandra Nicoletti, Michele Tinazzi

**Affiliations:** ^1^Section of Neurosciences, Department “G.F. Ingrassia”, University of Catania, Catania, Italy; ^2^Oasi Research Institute—IRCCS, Troina, Italy; ^3^Neurology Unit, Movement Disorders Division, Department of Neurosciences, Biomedicine and Movement Sciences, University of Verona, Verona, Italy; ^4^Center for Neurodegenerative Diseases (CEMAND), Department of Medicine, Surgery and Dentistry—Scuola Medica Salernitana, University of Salerno, Baronissi, Italy; ^5^Neurology Unit, Department of Clinical and Experimental Medicine, University of Pisa, Pisa, Italy; ^6^Department of Clinical and Experimental Sciences, University of Brescia, Brescia, Italy; ^7^FERB Onlus, Ospedale S. Isidoro, Bergamo, Italy; ^8^Parkinson and Movement Disorders Unit, Fondazione IRCCS Istituto Neurologico Carlo Besta, Milan, Italy; ^9^Department of Medical Area, Neurology Unit, ASST Pavia, Pavia, Italy; ^10^Botulinum Toxin Center, Neurology Unit A.O.U. Mater Domini, Catanzaro, Italy; ^11^Aldo Ravelli Research Center for Neurotechnology and Experimental Brain Therapeutics, Department of Health Sciences, University of Milan, Milan, Italy; ^12^IRCCS Neuromed, Pozzilli, Italy; ^13^Department of Medicine and Aging Sciences, University G. d'Annunzio, Chieti, Italy; ^14^Department of Medicine and Aging Sciences, University G. d'Annunzio, Pescara, Italy; ^15^IRCCS Humanitas Research Hospital, Milan, Italy; ^16^Department of Human Neurosciences, Università La Sapienza, Rome, Italy; ^17^Department of Advanced Medical and Surgery Sciences, University of Campania—Luigi Vanvitelli, Naples, Italy; ^18^Department of Neuroscience—Rita Levi Montalcini, University of Turin, Turin, Italy; ^19^Department of Biomedical and Neuromotor Sciences, University of Bologna, Bologna, Italy; ^20^IRCCS, Institute of Neurological Sciences of Bologna, Bologna, Italy; ^21^Movement Disorder Unit, Fondazione Policlinico Universitario A. Gemelli IRCCS, Rome, Italy; ^22^Neurosciences Research Centre, Molecular and Clinical Sciences Neurosciences Research Centre, Molecular and Clinical Sciences Research Institute, St George's University of London, London, United Kingdom; ^23^Department of Experimental and Clinical Medicine, University of Messina, Messina, Italy; ^24^Clinical Neurophysiology Unit, Cardarelli Hospital, Naples, Italy; ^25^Department of Neurosciences, Reproductive and Odontostomatological Sciences, University of Naples—Federico II, Naples, Italy; ^26^IRCCS Mondino Foundation, Pavia, Italy; ^27^Department of Brain and Behavioral Sciences, University of Pavia, Pavia, Italy; ^28^Clinical Neurology Unit, Department of Medical, Surgical and Health Services, University of Trieste, Trieste, Italy; ^29^University and Institute of Research and Medical Care San Raffaele, Rome, Italy; ^30^Ospedale degli Infermi, Biella, Italy; ^31^Psichiatria, Ospedale San Maurizio, Bolzano, Italy; ^32^Department of Medical Sciences and Public Health, University of Cagliari, Cagliari, Italy

**Keywords:** cluster analysis, clinical phenotypes, Functional Motor Disorders, data-driven phenotyping, functional neurological disorder

## Abstract

**Introduction:**

Functional Motor Disorders (FMDs) represent nosological entities with no clear phenotypic characterization, especially in patients with multiple (combined FMDs) motor manifestations. A data-driven approach using cluster analysis of clinical data has been proposed as an analytic method to obtain non-hierarchical unbiased classifications. The study aimed to identify clinical subtypes of combined FMDs using a data-driven approach to overcome possible limits related to “a priori” classifications and clinical overlapping.

**Methods:**

Data were obtained by the Italian Registry of Functional Motor Disorders. Patients identified with multiple or “combined” FMDs by standardized clinical assessments were selected to be analyzed. Non-hierarchical cluster analysis was performed based on FMDs phenomenology. Multivariate analysis was then performed after adjustment for principal confounding variables.

**Results:**

From a study population of *n* = 410 subjects with FMDs, we selected *n* = 188 subjects [women: 133 (70.7%); age: 47.9 ± 14.4 years; disease duration: 6.4 ± 7.7 years] presenting combined FMDs to be analyzed. Based on motor phenotype, two independent clusters were identified: Cluster C1 (*n* = 82; 43.6%) and Cluster C2 (*n* = 106; 56.4%). Cluster C1 was characterized by functional tremor plus parkinsonism as the main clinical phenotype. Cluster C2 mainly included subjects with functional weakness. Cluster C1 included older subjects suffering from anxiety who were more treated with botulinum toxin and antiepileptics. Cluster C2 included younger subjects referring to different associated symptoms, such as pain, headache, and visual disturbances, who were more treated with antidepressants.

**Conclusion:**

Using a data-driven approach of clinical data from the Italian registry, we differentiated clinical subtypes among combined FMDs to be validated by prospective studies.

## Introduction

Functional Motor Disorders (FMDs) still represent nosological entities with no clear phenotypic characterization (1, 2). To be defined, they require evidence of clinical inconsistency and incongruence in the context of functional weakness or hypo-/hyperkinesia. Once defined, FMD characterization is still principally based on main clinical features (1, 2). However, in most cases, patients may present a combined phenomenology of two or more FMDs, leading to a clinical classification that is usually based on the predominant symptom judged by the referring clinicians and then potentially biased (2).

We have recently observed that individuals with single, isolated FMD without comorbid neurological disorders have largely overlapping demographic and clinical non-motor characteristics (2). On the other hand, the characterization of multiple or “combined” FMDs remains still difficult, considering the variability related to the clinical judgment, as well as the temporal inconsistency of the disorder.

Data-driven approach using clustering analysis of clinical data has been proposed as an analytic method to obtain non-hierarchical, unbiased classifications to be tested in clinical practice. It has been applied to detect possible disease phenotypes in different fields of movement disorders, including Parkinson's disease (3) and essential tremor (4).

In this study, we aimed to identify possible clinical subtypes of combined FMDs using a data-driven approach, to overcome possible limits related to “*a priori*” classifications and clinical overlapping.

## Materials and methods

### Study population and data collection

Data were obtained by the Italian Registry of Functional Motor Disorders (IRFMD), managed by the University of Verona and the Italian Academy for the Study of Parkinson's Disease and Other Movement Disorders (Accademia LIMPE-DISMOV RADAC project) and Fondazione LIMPE. Details about subjects' recruitment and data collection have been described elsewhere (1, 2).

In brief, consecutive outpatients with FMDs were recruited from 25 tertiary movement disorders centers (11 in northern, five in central, six in southern Italy, and three in Sardinia/Sicily) between 1 September 2018 and 31 August 2019. Inclusion criteria were: age ≥10 years; the presence of one or more FMDs; a clinically definite diagnosis of FMDs based on Gupta and Lang diagnostic criteria (1, 2). Each patient with FMDs underwent a detailed clinical assessment, including screening for possible associated comorbidities. Patients with comorbid neurological diseases were included in the study (1, 5). FMD phenotypes were defined based on their specific phenomenological features in (a) tremor, (b) weakness, (c) dystonia; (d) myoclonus-like jerks; (e) facial motor disorders; (f) parkinsonism; and (g) gait disorders (1, 2). For functional weakness, we applied clues for the functional cause suggested by the referred Gupta and Lang diagnostic criteria, including the presence of distractibility maneuvers and the demonstration of positive signs (1). Since we focused on the symptom of functional weakness without selecting cases based on any etiological assumptions, no specific DSM criteria were applied for the diagnosis. Exclusion criteria were the presence of cognitive or physical impairment that precluded signing the informed consent form for participation in the study, as well as data acquisition from the structured interview (1).

Patients identified with one (isolated FMDs) or multiple FMDs (combined FMDs; e.g., dystonia plus tremor) underwent standardized clinical assessments. Patients were assessed at each center in a single session by a neurologist specialized in movement disorders. Demographic and clinical data were systematically collected, including associated symptoms, comorbidities, and practiced treatment (1, 2).

Approval was obtained by the Institutional Ethics Committee of the Coordinator Center (University of Verona, Azienda Ospedaliera Universitaria Integrata Verona, Prog. 1757CESC) and confirmed by the Committees of each participating center. All patients (or their guardians) gave their written consent to participate.

### Statistical analysis

Data are presented as mean ± standard deviation for continuous variables and frequency (percentage) for categorical variables.

Non-hierarchical cluster analysis using the *k-means* method was performed based on the main clinical phenomenology of FMDs: tremor, weakness, dystonia, myoclonus-like jerks, facial movement disorder, parkinsonism, and gait disorder (*n* = 7 variables). The Calinski-Harabasz pseudo-*F* stopping-rule criterion was used to evaluate the optimal number of clusters to be analyzed. We performed a cluster analysis by selecting the subgroup of patients with combined FMDs from the study population. The χ^2^ goodness of fit test was applied to determine if multicategorical variables distribution in the selected sample followed the expected distribution of the entire study population.

Differences in scalar variables among groups were tested using the independent-samples *t*-test, after testing for normality. Categorical variables were tested using the χ^2^*-*test. Unconditional logistic regression analysis was performed and for each study variable, we calculated Odds Ratios (OR), 95% Confidence Interval (CI), and *p-*value (two-tailed). Multivariate analysis was also performed after adjustment for principal confounding variables (age, sex, and disease duration). The significance level was set at *p* = 0.1.

## Results

We analyzed data from a total sample size of *n* = 410 enrolled subjects [women: 291 (71%); age 46.6 ± 15.8 years; disease duration: 5.6 ± 6.8 years)] presenting isolated and combined FMDs. FMDs phenomenology distribution in the study population was: tremor [*n* = 167 (40.7%)], weakness [*n* = 180 (43.9%)], dystonia [*n* = 119 (29%)], myoclonus [*n* = 53 (12.9%)], facial movement disorder [*n* = 47 (11.5%)], parkinsonism [*n* = 24 (5.8%)], and gait disorder [*n* = 109 (26.6%)]. Detailed descriptive analysis of demographic and clinical data of the study population has been already reported elsewhere (1).

A study subgroup of *n* = 188 (45.8%) subjects [women: 133 (70.7%); age: 47.9 ± 14.4 years; disease duration: 6.4 ± 7.7 years] with combined FMD was selected to be analyzed with data clustering. FMDs phenomenology distribution in this study subgroup was: tremor [*n* = 109 (58%)], weakness [*n* = 106 (56.4%)], dystonia [*n*= 78 (41.5%)], myoclonus [*n* = 35 (18.6%)], facial movement disorder [*n* = 35 (18.6%)], parkinsonism [*n* = 22 (11.7%)], and gait disorder [*n* = 92 (48.9%)]. There were no differences between the selected sample and the entire population in terms of age (*t* = 0.96; *p* = 0.337), sex (χ^2^ = 0.003; *p* = 0.954), and disease duration (*t* = 1.28; *p* = 0.201). The frequency distribution of overall FMDs differed from those in the entire study population (goodness of fit χ^2^ = 76.6; *p* < 0.001).

Two independent groups of patients based on clustering parameters were identified (for *n* = 2 to six clusters: *n* = 2 pseudo *F* = 45.34; *n* = 3 pseudo *F* = 40.16; *n* = 4 pseudo *F* = 36.68; *n* = 5 pseudo *F* = 31.27; *n* = *6* pseudo *F* = 34.66): Cluster C1 (*n* = 82; 43.6%) and Cluster C2 (*n* = 106; 56.4%). The distribution of FMDs among groups is shown in Table 1. Statistically significant differences in evaluated clinical variables between the two clusters are shown in Table 2, including multivariate analysis adjusted for age, sex, and disease duration.

**Table 1:** Distribution of FMDs among identified clusters.

**Combined FMDs**	**Cluster C1**	**Cluster C2**	**χ^2^ (*p*)**
	**(*n* = 82; 43.6%)**	**(*n* = 106; 56.4%)**	
Tremor	59 (71.9%)	50 (47.2%)	11.65 (< 0.001)^**^
Weakness	0	106 (100%)	188 (< 0.001)^**^
Dystonia	37 (45.1%)	41 (38.7%)	0.79 (0.374)
Myoclonus	19 (23.2%)	16 (15.1%)	1.99 (0.158)
Facial Movement Disorders	17 (20.7%)	18 (17%)	0.43 (0.512)
Parkinsonism	18 (21.9%)	4 (3.8%)	14.8 (< 0.001)^**^
Gait disorder	40 (48.8%)	52 (49.1%)	0.001 (0.97)

** *p* < 0.05.

FMDs, Functional Motor Disorders.

**Table 2:** Differences in evaluated clinical variables between the identified clusters.

**Combined FMDs^†^**	**Cluster C1**	**Cluster C2**	**OR (95% CI)**	***p***	**adjOR (95% CI)**	***p***
Age, years	52.1 ± 13.6	44.8 ± 14.2	0.96 (0.94–0.98)	0.001**	/	/
Sex, male	29 (35.4%)	26 (24.5%)	0.59 (0.31–1.11)	0.107	/	/
Disease duration, years	6.4 ± 8.3	6.5 ± 7.3	1 (0.96–1.04)	0.967	/	/
Education, years	11.9 ± 4.1	11.6 ± 3.7	0.98 (0.9–1.06)	0.578	/	/
**Associated NMS**						
Pain	36 (49.3%)	68 (70.1%)	2.41 (1.28–4.53)	0.006**	1.88 (0.96–3.67)	0.064*
Headache	20 (27.4%)	40 (41.2%)	1.86 (0.97–3.58)	0.063*	1.79 (0.91–3.53)	0.093*
Dep. / Der.	5 (6.8%)	16 (16.5%)	2.69 (0.94–7.71)	0.066*	2.1 (0.71–6.25)	0.180
Anxiety	53 (72.6%)	57 (58.8%)	0.54 (0.28–1.03)	0.063*	0.5 (0.25–1)	0.051*
**Associated FD**						
Visual FS	5 (14.7%)	26 (40.7%)	3.97 (1.36–11.59)	0.012**	2.89 (0.93–9.03)	0.066*
**Comorbidities**						
PD and parkinsonism	6 (28.6%)	1 (4.3%)	0.11 (0.01–1.04)	0.054*	0.13 (0.01–1.58)	0.110
**Therapies**						
BTX Therapy	15 (42.9%)	10 (18.9%)	0.31 (0.12–0.81)	0.017**	0.31 (0.11–0.84)	0.021**
Antidepressants	26 (54.2%)	49 (73.1%)	2.3 (1.05–5.04)	0.037**	2.29 (1.01–5.19)	0.047**
Antiepileptics	20 (41.7%)	18 (26.9%)	0.51 (0.23–1.13)	0.098*	0.51 (0.22–1.14)	0.099*

**p < 0.05.

*p < 0.1.

Cluster C1 set as reference; adjusted OR (adjOR) with 95% Confidence Interval (95% CI) for age, sex, and disease duration.

FMDs, Functional Motor Disorders, NMS, Non-Motor Symptoms; Dep./Der., Depersonalization/Derealization; FD, Functional Disorders; FS, Functional Symptom; PD, Parkinson's Disease; BTX, Botulinum Toxin.

^†^Combined FMD: n = 188.

As for the total population, the first detected Cluster C1 principally included subjects with functional tremor *plus* parkinsonism as the main FMDs clinical phenotype. The tremor was present in *n* = 59 (71.9%) patients: in *n* = 49 (83%) patients, it was distributed in the upper limb, in *n* = 18 (30.5%) patients in the lower limb, and in *n* = 15 (25.4%) patients in body parts other than limbs. Parkinsonism was instead present in *n* = 18 (21.9%) patients: in *n* = 15 (83.3%) patients, it was distributed in the upper limb, in *n* = 8 (44.4%) patients in the lower limb, and in *n* = 10 (55.6%) patients in body parts other than limbs.

Functional weakness was instead more represented in Cluster C2, being present in all *n* = 106 patients. In *n* = 42 (39.6%) patients, it was distributed in the upper limb, in *n* = 86 (81.1%) patients in the lower limb, and in *n* = 12 (11.3%) patients in body parts other than limbs. In Cluster C2, parkinsonism was present in *n* = 4 (3.8%) patients: in *n* = 3 (75%) patients, it was distributed in the upper limb, in *n* = 3 (75%) patients in the lower limb, and in *n* = 3 (75%) patients in body parts other than limbs. In all patients affected by parkinsonism, functional weakness principally affecting limbs was observed. Other hyperkinetic functional movement disorders were equally distributed in the two clusters.

Cluster C1 included older subjects with associated anxiety. They presented a diagnosis of parkinsonism as neurological comorbidity, even if the association was lacking in the multivariate analysis, and they were more treated with BTX and antiepileptics. Cluster C2 included instead younger subjects referring to more associated symptoms, such as pain, headache, visual disturbances, and feelings of depersonalization/derealization, even though this last factor lacked a significant association in the multivariate analysis. They were treated with antidepressants with respect to Cluster C1.

## Discussion

In this study, we aimed to identify possible clinical subtypes of combined FMDs by clustering registry-based data. We used patients' main clinical features as clustering variables, then we looked at differences in terms of comorbidities associated with FMDs (5), precipitating and associated factors, as well as response to therapies among the identified groups.

When cluster analysis was applied to main clinical features with no “*a priori*” distinction on combined FMDs, two separate clusters were identified with different characteristics. The first cluster (Cluster C1) principally included subjects with functional movement disorders, specifically functional tremor *plus* parkinsonism. It also mainly included older subjects experiencing anxiety. They presented a diagnosis of parkinsonism as neurological comorbidity and they were treated with BTX and antiepileptics. The second cluster (Cluster C2) was characterized by weakness as the main clinical phenotype. The group principally included younger subjects referring to different associated symptoms, including headache, debilitating pain, visual symptoms, and feelings of depersonalization/derealization. Subjects in this group were more treated with antidepressants.

The high frequency of functional parkinsonism and tremor in Cluster C1 may reflect previous results showing that functional tremor represents one of the most frequent FMDs observable in elderly subjects (6). No specific differences in terms of gender distribution were obtained between the two groups, even though Cluster C1 included overall more men than Cluster C2. Previous reports highlighted a higher prevalence of conversion symptoms, including functional weakness and sensory symptoms in women with respect to men, probably explained by a higher rate of consultation for the referred condition in this group (7–9).

Concerning differences in associated conditions between groups, highlighted characteristics for Cluster C2 seemed in agreement with the previously reported association between functional weakness and sensory symptoms with respect to other phenotypes (1, 7, 8). By a pathophysiological state point, it has been argued that such results may be explained by the hypoactivity of subcortical circuits controlling sensorimotor function and driving both voluntary motor behavior and sensory loss, differently from what is hypothesized for functional symptoms characterized by excessive motor output (1, 10).

We also found a high prevalence of feelings of depersonalization/derealization in Cluster C2, in which functional weakness is more represented, with respect to Cluster C1. This may reflect a previous hypothesis postulating that functional weakness could be a form of “hemi-depersonalization” in which the relationship with the functional weakness could be explained in terms of immobility and/or increased attention paid to a specific body part (11).

Coexisting neurological comorbidities in FMDs, including parkinsonism, were already described in our study population (5). Patients with comorbid FMDs were usually older and more frequently had tremors (5), characteristics observed in Cluster C1. Systematic review already demonstrated that tremor is the most common FMD observed in Parkinson's disease, usually occurring in the most affected side (5, 12). It has been suggested that the underlying pathological process in Parkinson's disease predisposes patients to abnormal attention-to-movement production (12). The same abnormal attention toward movement has been demonstrated in FMD, a notion supported by the clinical evidence of functional movement normalization by shifting attention away from movement (12). Based on this assumption, it could be argued that Parkinson's disease pathology may produce vulnerability to develop functional symptoms. In the case of functional symptoms in the context of Parkinson's disease, they would be mainly motor symptoms linked phenomenologically to the physical deficit caused by the disease (12).

Our study has some limitations that should be considered. Selection bias cannot be excluded since the enrollment was carried out in tertiary, referral centers and collected data may be also biased by recall bias. Furthermore, data clustering was applied just to the combined FMDs, thus affecting the results generalizability. However, it should be underlined that the exclusion of the “isolated” FMDs from the data clustering was due to methodological limits. Indeed, the stopping-rule method could not be applied to identify an optimal number of clusters in the subpopulation of isolated FMDs because the optimal number of identifiable clusters overlaps with the number of the identified FMDs phenotypes used for the motor classification, as previously defined.

There are also strengths of the present study. This is the first study attempting an unbiased, data-driven classification of FMDs when combined, to overcome possible evaluation bias due to the subjective “a priori” classification. It also includes a large multicenter sample of FMDs patients which were systematically evaluated by a standardized protocol.

In conclusion, using a data-driven approach of clinical data from the Italian registry, we were able to detect clinical subtypes of combined FMDs to be validated by prospective studies. In future studies, it will be crucial to expand the ambition of diagnostic quality to postmortem studies.

## Data availability statement

The original contributions presented in the study are included in the article/supplementary material, further inquiries can be directed to the corresponding authors.

## Ethics statement

The studies involving human participants were reviewed and approved by University of Verona, Azienda Ospedaliera Universitaria Integrata Verona, Prog. 1757CESC. Written informed consent to participate in this study was provided by the participants' legal guardian/next of kin.

## Author contributions

GM: study design, data collection, data and statistical analysis, writing of the first draft, editing, and final draft. CG, AL, MZa, MT, and REr: study design, data collection, review, critique, editing, and final draft. AN: study design, data collection, data and statistical analysis, writing of the first draft, review, critique, editing, and final draft. EM, PBa, RC, SM, APil, APa, LR, REl, CD, CA, FB, PAB, BD, OG, NM, EO, LB, AA, GF, RD, MZi, GC-B, MP, FM, ME, APis, PM, FS, MCM, IDV, LT, FDB, TE, and GD: study design, data collection, review, and critique. All authors contributed to the article and approved the submitted version.

## Conflict of interest

The authors declare that the research was conducted in the absence of any commercial or financial relationships that could be construed as a potential conflict of interest.

## Publisher's note

All claims expressed in this article are solely those of the authors and do not necessarily represent those of their affiliated organizations, or those of the publisher, the editors and the reviewers. Any product that may be evaluated in this article, or claim that may be made by its manufacturer, is not guaranteed or endorsed by the publisher.

## Appendix

**Table A1:** Co-investigators.

**Name**	**Location**	**Role**	**Contribution**
Marialuisa Gandolfi MD, PhD	Department of Neurosciences, Biomedicine and Movement Sciences, University of Verona, Verona, Italy.	Site Investigator	Site coordinator of data acquisition
Sofia Cuoco, PhD	Center for Neurodegenerative Diseases (CEMAND) Department of Medicine, Surgery and Dentistry—Scuola Medica Salernitana, University of Salerno, Baronissi (Sa), Italy.	Site Investigator	Site coordinator of data acquisition
Sara Scannapieco, MD	Center for Neurodegenerative Diseases (CEMAND) Department of Medicine, Surgery and Dentistry—Scuola Medica Salernitana, University of Salerno, Baronissi (Sa), Italy.	Site Investigator	Site coordinator of data acquisition
Daniela Frosini, MD	Neurology Unit, Department of Clinical and Experimental Medicine, University of Pisa, Pisa, Italy.	Site Investigator	Site coordinator of data acquisition
Eleonora Del Prete, MD	Neurology Unit, Department of Clinical and Experimental Medicine, University of Pisa, Pisa, Italy.	Site Investigator	Site coordinator of data acquisition
Andrea Scalvini, MD	Department of Clinical and Experimental Sciences, University of Brescia, Brescia, Italy.	Site Investigator	Site coordinator of data acquisition
Alberto Imariso, MD	Department of Clinical and Experimental Sciences, University of Brescia, Brescia, Italy.	Site Investigator	Site coordinator of data acquisition
Antonio Emanuele Elia, MD	Parkinson and Movement Disorders Unit, Fondazione IRCCS Istituto Neurologico Carlo Besta, Milan, Italy.	Site Investigator	Site coordinator of data acquisition
Nico Golfrè Andreasi, MD	Parkinson and Movement Disorders Unit, Fondazione IRCCS Istituto Neurologico Carlo Besta, Milan, Italy.	Site Investigator	Site coordinator of data acquisition
Calogero Edoardo Cicero, MD, PhD	Department G.F. Ingrassia, Section of Neurosciences, University of Catania, Catania, Italy.	Site Investigator	Site coordinator of data acquisition
Sara Mazza, MD	Department of Medical Area, Neurology Unit, ASST Pavia, Pavia, Italy.	Site Investigator	Site coordinator of data acquisition
Gabriele Bellavia, MD	Department Of Medical Area, Neurology Unit, ASST Pavia, Pavia, Italy.	Site Investigator	Site coordinator of data acquisition
Marilisa Pasquale, MD	Botulinum Toxin Center, Neurology Unit A.O.U. Mater Domini, Catanzaro, Italy.	Site Investigator	Site coordinator of data acquisition
Giorgio Spano, MD	Botulinum Toxin Center, Neurology Unit A.O.U. Mater Domini, Catanzaro, Italy.	Site Investigator	Site coordinator of data acquisition
Alberto Priori MD, PhD	Aldo Ravelli Research Center For Neurotechnology and Experimental Brain Therapeutics, Department of Health Sciences, University of Milan, Milan Italy.	Site Investigator	Site coordinator of data acquisition
Cinzia Femiano, MD	IRCCS Neuromed, Pozzilli, Italy.	Site Investigator	Site coordinator of data acquisition
Giada Ricciardo Rizzo, MD PhD	IRCCS Neuromed, Pozzilli, Italy.	Site Investigator	Coordinated acquisition of data for site
Marco Onofrj, MD	Department of Neuroscience, Imaging And Clinical Sciences -University G. D'annunzio, Chieti-Pescara, Italy.	Site Investigator	Site coordinator of data acquisition
Stefania Lalli, MD, PhD	Department of Neurology, IRCCS Humanitas Research Hospital, Rozzano, Milan, Italy.	Site Investigator	Site coordinator of data acquisition
Giovanni Fabbrini, MD	Department of Human Neurosciences, La Sapienza, University of Rome, Rome, Italy. IRCCS Neuromed, Pozzilli, Italy.	Site Investigator	Site coordinator of data acquisition
Alessandro Tessitore MD, Ph.D.	Department of Advanced Medical and Surgery Sciences, University of Campania—Luigi Vanvitelli, Naples, Italy.	Site Investigator	Site coordinator of data acquisition
Leonardo Lopiano, MD, PhD	Department of Neuroscience—Rita Levi Montalcini, University of Turin, Turin, Italy.	Site Investigator	Site coordinator of data acquisition
Luisa Sambati MD, PhD	IRCCS Istituto delle Scienze Neurologiche di Bologna, Bologna, Italia.	Site Investigator	Site coordinator of data acquisition
Anna Rita Bentivoglio, MD, PhD	Institute of Neurology, Movement Disorder Research Center, Università Cattolica del Sacro Cuore; Movement Disorder Unit, Fondazione Policlinico Universitario A. Gemelli IRCCS, Rome, Italy.	Site Investigator	Site coordinator of data acquisition
Giulia Di Lazzaro, MD	Department Systems Medicine, University of Rome Tor Vergata, Rome, Italy.	Site Investigator	Site coordinator of data acquisition
Giulia Bellavita, MD	Clinical Neurology Unit, Department of Medical, Surgical and Health Services, University of Trieste, Italy.	Site Investigator	Site coordinator of data acquisition
Paola Caruso, MD	Clinical Neurology Unit, Department of Medical, Surgical and Health Services, University of Trieste, Italy.	Site Investigator	Site coordinator of data acquisition

Italian Registry of Functional Motor Disorders (IRFMDs) study group.
